# Artificial Intelligence Applications for COVID-19 in Intensive Care and Emergency Settings: A Systematic Review

**DOI:** 10.3390/ijerph18094749

**Published:** 2021-04-29

**Authors:** Marcel Lucas Chee, Marcus Eng Hock Ong, Fahad Javaid Siddiqui, Zhongheng Zhang, Shir Lynn Lim, Andrew Fu Wah Ho, Nan Liu

**Affiliations:** 1 Faculty of Medicine, Nursing and Health Sciences, Monash University, Victoria 3800, Australia; tche0014@student.monash.edu; 2 Duke-NUS Medical School, National University of Singapore, Singapore 169857, Singapore; marcus.ong.e.h@singhealth.com.sg (M.E.H.O.); fahad.siddiqui@duke-nus.edu.sg (F.J.S.); andrew.ho@mohh.com.sg (A.F.W.H.); 3 Department of Emergency Medicine, Singapore General Hospital, Singapore 169608, Singapore; 4 Department of Emergency Medicine, Sir Run Run Shaw Hospital, Zhejiang University School of Medicine, Hangzhou 310016, China; zh_zhang1984@zju.edu.cn; 5 Department of Cardiology, National University Heart Centre, Singapore 119074, Singapore; shir_lynn_lim@nuhs.edu.sg; 6 Health Service Research Centre, Singapore Health Services, Singapore 169856, Singapore; 7 Institute of Data Science, National University of Singapore, Singapore 117602, Singapore

**Keywords:** artificial intelligence, machine learning, COVID-19, emergency department, intensive care, critical care

## Abstract

*Background*: Little is known about the role of artificial intelligence (AI) as a decisive technology in the clinical management of COVID-19 patients. We aimed to systematically review and critically appraise the current evidence on AI applications for COVID-19 in intensive care and emergency settings. *Methods*: We systematically searched PubMed, Embase, Scopus, CINAHL, IEEE Xplore, and ACM Digital Library databases from inception to 1 October 2020, without language restrictions. We included peer-reviewed original studies that applied AI for COVID-19 patients, healthcare workers, or health systems in intensive care, emergency, or prehospital settings. We assessed predictive modelling studies and critically appraised the methodology and key findings of all other studies. *Results*: Of fourteen eligible studies, eleven developed prognostic or diagnostic AI predictive models, all of which were assessed to be at high risk of bias. Common pitfalls included inadequate sample sizes, poor handling of missing data, failure to account for censored participants, and weak validation of models. *Conclusions*: Current AI applications for COVID-19 are not ready for deployment in acute care settings, given their limited scope and poor quality. Our findings underscore the need for improvements to facilitate safe and effective clinical adoption of AI applications, for and beyond the COVID-19 pandemic.

## 1. Introduction

The ongoing coronavirus disease 2019 (COVID-19) pandemic has challenged healthcare systems and healthcare practitioners worldwide. Intensive care units (ICU) and emergency departments (ED) in badly afflicted areas have been overwhelmed by the surge in patients suspected or diagnosed with COVID-19 [1,2,3]. This exerts significant pressure on healthcare resources, necessitating novel diagnostic and care pathways to rationally deploy scarce emergency and intensive care healthcare resources. Current strategies and recommendations on clinical management and resource rationalisation draw on past pandemic experiences and expert recommendations [3,4,5]; however, there has been growing interest in novel applications of artificial intelligence (AI) to assist in the COVID-19 response within these settings.

AI is a branch of computer science that uses computational methods to mimic human intelligence. AI is becoming increasingly ubiquitous; from self-driving cars to Siri, drone-assisted farming to diagnosis, AI applications have become indispensable in many industries, including the healthcare sector. Exponential advances in computer processing speeds, increased access to big data, and electronic medical record usage have all been crucial factors in driving the uptake of AI applications within the medical field. In medical research, AI often refers specifically to machine learning, which is a subset of AI that focuses on automatic improvement of computer programmes through experience [6,7]. For example, simple forms of machine learning such as logistic, linear, or Cox regression have commonly been used to investigate associations between predictors and disease outcomes. More advanced machine learning models, including random forest models, neural networks, or support vector machines, are also becoming more common in the medical literature. Such models can assist with more complex tasks, for example, image analysis and diagnosis in radiology [8], dermatology [9], and ophthalmology [10], amongst others. In this review, AI is not limited to machine learning models but includes broader forms of AI such as natural language processing, decision trees, and computational decision assistance software. In pre-COVID-19 intensive care and emergency settings, AI applications have assisted with automated patient monitoring [11,12,13,14], prognostication [15], and optimisation of staffing allocations [16,17,18,19]. Given the unprecedented volume of COVID-19 patients, recent reviews have also identified resource optimisation of ICU beds as a potentially significant application of AI [20,21].

Earlier systematic reviews have identified significant issues in the quality and reporting of predictive models for COVID-19 diagnosis and prognosis [22] and AI applications for classifying COVID-19 medical images [23]. Shillian, et al. [24] in a systematic review of machine learning studies in pre-COVID-19 ICUs reported similar issues, such as limited sample size and poor validation of predictions. However, no study has evaluated the scope and quality of all available AI applications for COVID-19 in intensive care and emergency settings. We hypothesise that issues in quality and reporting will exist across all AI applications in these settings due to the urgency and limited time afforded to developing these applications during pandemic. However, we also anticipate that diverse, pre-COVID-19 applications of AI may have been repurposed for use in the COVID-19 pandemic, including diagnosis, prognostication, monitoring, and resource optimisation. An exploration of the quality and breadth of current AI applications will provide valuable insight for improving the development and deployment of AI applications in intensive care and emergency settings, for and beyond the COVID-19 pandemic. We aim to systematically review and critically appraise the current evidence on AI applications for COVID-19 in intensive care and emergency settings, focussing on methods, reporting standards, and clinical utility.

## 2. Materials and Methods

We reported this systematic review according to the Preferred Reporting Items for Systematic Reviews (PRISMA) guidelines (Supplementary File S1). A review protocol was developed but was not publicly registered.

### 2.1. Search Strategy and Selection Criteria

We searched six databases: (PubMed, Embase, Scopus, CINAHL, IEEE Xplore, and ACM Digital Library) by combining search terms related to AI, COVID-19, and intensive care or emergency settings. PubMed, Embase, Scopus, and CINAHL were chosen for their broad coverage across biomedical, nursing, allied health, and general scientific literature, while IEEE Xplore and ACM Digital Library were included for coverage of more technical AI literature. For brevity, the truncated search strategy showing only the first three terms in each concept set is as follows: ((“Artificial intelligence” OR “Deep learning” OR “Machine learning” OR …) AND (“COVID-19” OR “Coronavirus disease 2019” OR “2019-nCoV” OR …) AND (Emergency OR “ED” OR “intensive care” OR …)). The complete search strategy can be found in Supplementary File S2. We also screened the reference lists of included articles to identify additional relevant studies.

We included articles that met the following criteria: (1) applied AI; (2) investigated COVID-19 operations of ICU, ED, or emergency medical services (EMS) or analysed data from COVID-19 patients in the ED or within a prehospital setting, COVID-19 patients requiring intensive care (admission to the ICU, mechanical ventilation, or a composite including either of these outcomes), or the healthcare workers treating these patients, including ED or ICU physicians and nurses as well as paramedics; and (3) were original, peer-reviewed research articles. For this review, only conventional machine learning algorithms such as random forest models, neural networks, or support vector machines were considered AI; multivariable logistic regression predictive models (including ridge and least absolute shrinkage and selection operator (LASSO) regression) were excluded. No restrictions were placed on the language of articles; all non-English articles that appeared in our search were translated and assessed for suitability, however, we did not search the databases using equivalent non-English search terms.

### 2.2. Literature Selection and Data Extraction

We conducted an initial search on 30 August 2020 and updated the results on 1 October 2020. Articles were screened by title, abstract, and, if ambiguous, full text by two independent reviewers (MLC and NL). Subsequently, the two reviewers (MLC and NL) independently extracted data using a standardised data extraction form. Discrepancies in article selection and data extraction were resolved between reviewers through discussion.

We extracted the following data for all included articles: country of study population, outcome predicted, sample size of the training and validation datasets, AI algorithms used, discrimination (e.g., C-index, accuracy) and calibration (e.g., calibration slope, Brier loss score) of models on the strictest form of validation, features included in the final model, and transparent reporting of a multivariable prediction model for individual prognosis or diagnosis (TRIPOD) study type [25], if applicable.

### 2.3. Data Analysis

For studies including multivariate AI predictive models, we evaluated the risk of bias within the study methodology using prediction model risk of bias assessment tool (PROBAST) [26]. PROBAST is a structured tool comprising 20 signalling questions for assessing the risk of bias and applicability across the four domains of participants, predictors, outcome, and analysis. Applicability of included studies was not assessed as our study was not concerned with a specific application of AI predictive models. In lieu of specific reporting standards for AI studies at the time of study conception [27], we assessed the reporting quality of multivariable predictive modelling studies using an adaptation of Wang, et al. [28] modified TRIPOD statement [29] for AI models (Supplementary File S3). The TRIPOD statement is a reporting guideline for studies that develop, validate, or update a prediction model, often for diagnostic or prognostic purposes. It contains 22 checklist items for assessing the reporting quality of predictive modelling studies. For studies that could not be assessed using the above guidelines (for example, non-predictive modelling studies), we summarised the study methodology, including data sources, application of AI, and validation methods, as well as the key findings of the study.

## 3. Results

### 3.1. Study Characteristics

**Figure 1:**
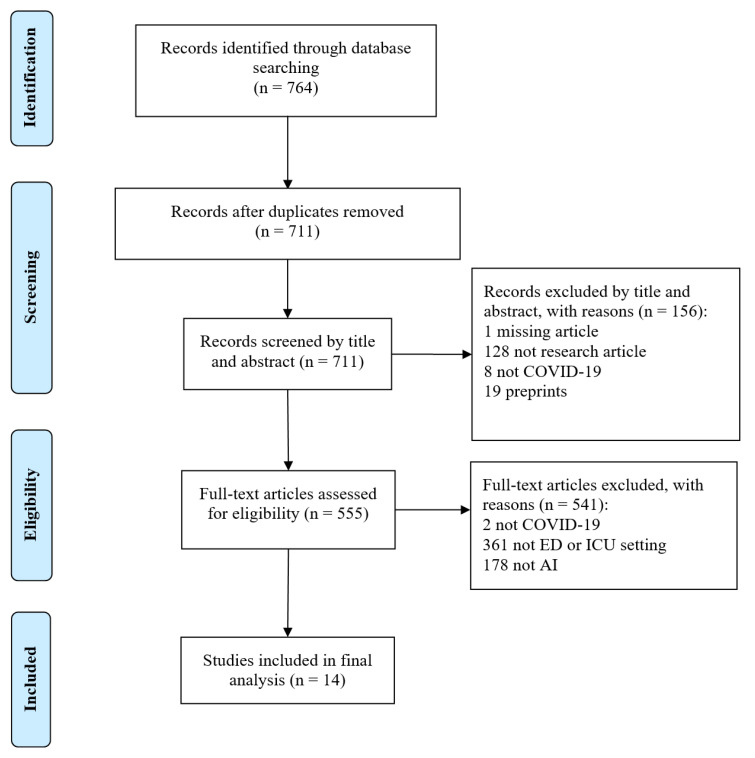
PRISMA flow diagram.

Our search of the six databases returned 764 studies, of which 14 were included and analysed in this review (Figure 1). Table 1 presents the main characteristics of the study. Eleven of the 14 studies investigated predictive models and were assessed according to PROBAST and TRIPOD: eight studies developed prognostic models [30,31,32,33,34,35,36,37] and three studies developed diagnostic models [38,39,40]. Of the remaining three studies, two evaluated the prognostic potential of existing AI-based lung segmentation software (without integration into a multivariate predictive model) [41,42] and one investigated an AI-based system for resource optimisation in the ICU [43]. Eleven studies used patient data collected from the ICU and four studies used data from the ED. No study collected data from the prehospital setting, despite including prehospital-related search terms in the search strategy.

In terms of country of study, Italy (*n* = 3) and United States (*n* = 3) were represented by more than one study, while Brazil, Canada, China, France, Germany, Israel, Turkey, and the United Kingdom had one study each.

According to the TRIPOD classification of predictive models, two studies were classified as Type 2b (validation using a non-random split of data by time and/or location), three studies as Type 2a (validation using a random split of data such as a train-test split), four studies as Type 1b (validation using re-sampling techniques such as bootstrapping or k-fold cross-validation), and one study as Type 1a (no validation, only evaluation of apparent model performance on the same training dataset). One study that conducted development and validation using data from separate studies was considered Type 3.

### 3.2. Risk of Bias

Table 2 presents the risk of bias assessment of AI predictive models according to PROBAST. All 11 predictive modelling studies had a high overall risk of bias. Two out of 11 studies had an unclear risk of bias within the participant domain. Unclear risk of bias in the participant domain was mainly due to ambiguous exclusion criteria that may lead to the study population not being representative of the intended target population [36,37].

All three studies at a high risk of bias in the predictor domain were prognostic. Two studies [35,36] used retrospective, multicentre data and were at risk of bias from varying methods of predictor assessment at different centres. The remaining study [34] obtained predictor data from the most recent assessments available, instead of assessing predictors at the intended time of use. Two studies did not report adequately on the assessment of computed tomography (CT) [39] or other features [37], resulting in an unclear risk of bias.

Two and four out of 11 studies were at high and unclear risk of bias within the outcome domain, respectively. In many prognostic studies [31,32,34,37], the criteria for ICU admission and blinding of outcome determination to predictor variables were often not reported, leading to an unclear risk of bias.

Within the analysis domain, all eleven studies had insufficient outcome events per variable (EPV) (<20 EPV for model development studies and <100 for model validation studies) leading to a high risk of bias. Furthermore, no study reported on model calibration and only two studies [37,38] appropriately handled and reported on missing data. Prognostic predictive models were particularly at risk of inadequately accounting for, or reporting on, censored patients who were still hospitalised without the outcome (e.g., ICU admission) at the end of the study period. Only one study appropriately accounted for censored data by combining deep learning techniques with traditional Cox regression [36].

### 3.3. Adherence to Reporting Standards

**Figure 2:**
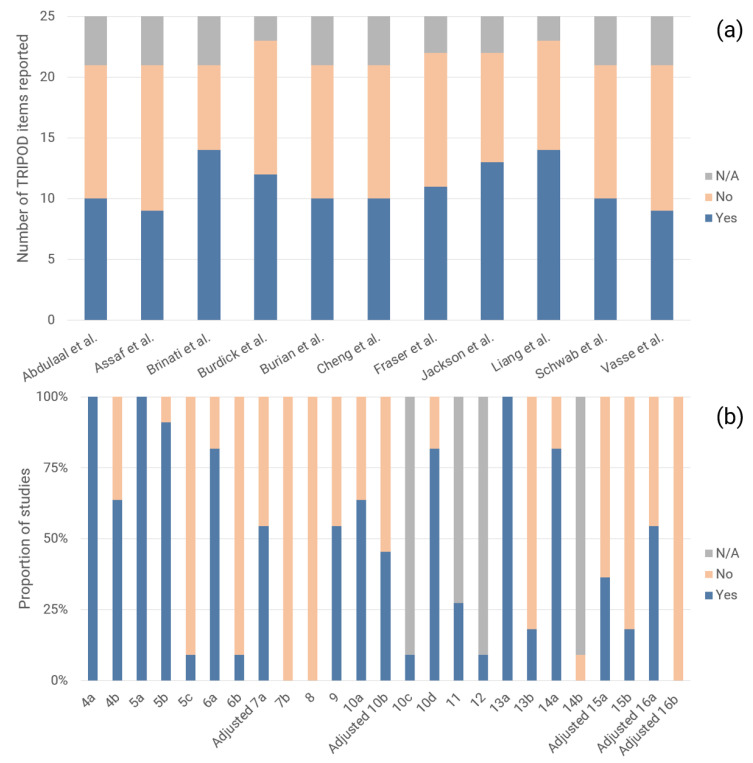
(**a**) Number of TRIPOD items reported per study and (**b**) Proportion of studies reporting on each TRIPOD item.

The modified TRIPOD checklist comprised 25 terms, including 17 terms for reporting of methods and eight terms for results. Figure 2 describes the adherence of studies to reporting standards, as assessed by the modified TRIPOD checklist. Studies reported on a median of 48% (IQR: 48–59%) of relevant TRIPOD items, with 10 of 25 TRIPOD items having 50% adherence or less. Additionally, the following eight TRIPOD items had 25% adherence or less: reporting on treatments administered to study participants (item 5c), blinding of outcome and predictor assessment (items 6b and 7b), study size determination (item 8), reporting on characteristics of study participants, including proportions of participants with missing data (item 13b), reporting of unadjusted associations between predictors and outcomes in multivariable logistic regression models (item 14b), explanation of how to use the prediction model (item 15b), and calibration and method of calibration (adjusted item 16b).

### 3.4. Diagnosis

Three studies investigated diagnostic AI predictive models; two studies developed models to predict the outcome of COVID-19 status at admission to the ED. Only one study was externally validated: Vasse, et al. [40] developed a decision tree based on cellular population data using random forest for feature selection (accuracy = 60.5%). Brinati, et al.’s [38] random forest model (C-index = 0.84, accuracy = 82%) and three-way random forest model (accuracy = 86%) achieved better performance, but were validated using weaker *k*-fold cross-validation. Both studies included leucocyte or a leucocyte sub-population count as a predictor in their final model.

The third study [39] developed a decision tree for determining COVID-19 infection status in the ICU based on plasma inflammatory analyte features selected by a random forest classifier. On five-fold cross-validation, this classifier achieved an accuracy of 98%.

### 3.5. Prognosis

Most studies on prognostic AI predictive models (9/10, 90%) predicted ICU admission, mechanical ventilation, or a similar composite outcome of severe or critical illness. Collectively, such studies reported C-indices between 0.79 and 0.98. Liang, et al.’s [36] deep learning survival Cox model had the largest training cohort of 1590 patients and achieved a C-index of 0.890, 0.852, and 0.967 when externally validated on cohorts of 801, 305, and 73 patients from Wuhan, Hubei, and Guangzhou, respectively. Schwab et al.’s [37] support vector machine achieved a superior C-index of 0.98 on a weaker internal validation and a smaller sample size for testing model performance.

The artificial neural network trained by Abdulaal, et al. [30] using data collected at ED admission (C-index = 0.901) was the only prognostic AI model developed to predict in-hospital mortality in COVID-19 patients.

Apart from predictive modelling, Durhan et al. [41] and Mushtaq et al. [42] evaluated the prognostic utility of two separate deep learning-based software that determine the normal lung proportion and total lung involvement, respectively. Scores obtained from each software achieved a C-index of 0.944 and 0.77 for predicting ICU admission, respectively. While multivariate predictive models were not developed, both studies were subject to similar issues in development and reporting, including ambiguous criteria for ICU admission, inappropriate handling of missing data using complete-case analysis, and lack of reporting on treatments received by participants and on blinding of the outcome.

### 3.6. Other Applications

Apart from diagnostic and prognostic applications, Belciug, et al. [43] utilised an artificial immune system algorithm, a type of evolutionary AI algorithm, to optimise a queueing model for simulating hospital bed allocation in the ICU. The final model, intended as a tool for hospital managers, proposes an optimal admission rate and number of beds while balancing the costs associated with increasing capacity and refusing patients. The model was applied to ICU data published by the Ministry of Health of Italy and estimated a minimum rejection rate of 3.4% and 1.7% of patients requiring ICU admission from 13 March 2020 to 23 March 2020 (average daily volume of 200 patients) and 23 March 2020 to 30 March 2020 (average daily volume of 63 patients), respectively. However, these estimates were not validated.

## 4. Discussion

Our study is the first systematic review of AI applications for COVID-19 in intensive care and emergency settings. Applications were largely limited to diagnostic and prognostic predictive modelling, with only one study investigating a separate application of simulating ICU bed occupancy for resource optimisation. Due to high risk of bias, inadequate validation, or poor adherence to reporting standards in all reviewed studies, we have found no AI application for COVID-19 ready for clinical deployment in intensive care or emergency settings.

Among the reviewed articles, we found a limited range of AI applications being studied within intensive care and emergency settings. An exploratory review identified early detection and diagnosis, resource management of hospital beds or healthcare workers, and automatic monitoring and prognostication as possible applications of AI for the COVID-19 pandemic [20]. However, current applications within the reviewed articles mainly comprised prognostic models for critical illness or diagnostic models to predict COVID-19 status, none of which are ready for clinical use. Only one preliminary study by Belciug et al. [43], which lacked validation, investigated allocative simulation and resource optimisation in the ICU, while no study investigated automatic monitoring or prognostication of COVID-19 patients. Belciug, et al. studied ICU resource optimisation employing queueing theory, a mathematical field of study, and artificial immune systems, an evolutionary AI algorithm that is uncommonly utilised in medical research. Unfamiliarity and the absence of general adoption of these methods within the medical community may contribute to the paucity of studies exploring less common but potentially impactful AI applications. As highlighted in previous literature [22,44], robust interdisciplinary collaboration and communication will be crucial in stimulating broader applications of AI for COVID-19 in intensive care and emergency settings, as well as the in medical literature at large.

Assessment of AI predictive models also revealed significant deficiencies in model development, validation, and reporting. These findings corroborate with earlier systematic reviews on predictive models for COVID-19 [22] and in intensive care settings [24]. Studies developing AI models should adhere to the TRIPOD reporting guidelines [25], PROBAST [26], or, ideally, recent AI-specific guidelines. These include the guidelines for transparency, reproducibility, ethics, and effectiveness (TREE) [45], Consolidated Standards of Reporting Trials-Artificial Intelligence (CONSORT-AI) [46], and Standard Protocol Items: Recommendations for Interventional Trials-Artificial Intelligence (SPIRIT-AI) [47]. While the above guidelines provide comprehensive explanations and elaborations, we emphasise hereinafter several common problematic areas within the reviewed studies and recommendations for future studies.

The most common source of bias was an inadequate sample size, which was found in all studies. A low sample size introduces the risk of over-fitting and model optimism. A benchmark for the development of logistic regression models is 20 EPV [4,26,48], while models using AI algorithms like random forest, support vector machines, and neural networks may require up to 200 EPV to account for model optimism [49]; a minimum of 100 EPV is recommended for validation studies [26]. Missing data also contributed significantly to bias; only two studies appropriately handled and reported on missing data. Ideally, the proportion of missing data for each variable should be reported [25] and multiple imputation should be used to avoid bias from inappropriate exclusion of participants with missing data (i.e., complete-case analysis) [50,51,52,53]. However, if complete-case analysis is used, authors should provide a comparative analysis of model performance with and without excluded participants to facilitate the judgement of bias from exclusion. For prognostic studies, studies often failed to appropriately account for censored patients (e.g., neither discharged nor admitted to the ICU). Censored patients should be handled using a time-to-event analysis such as Cox regression; inappropriate exclusion of these patients may lead to a skewed dataset that includes fewer patients without the outcome, introducing bias into the model [26]. For diagnostic studies, bias was often introduced by using the reverse transcription-polymerase chain reaction (RT-PCR) test as the ground truth or gold-standard for COVID-19 diagnosis, despite potentially poor sensitivity [54]. We recommend repeat RT-PCR testing to minimise the likelihood of false-negative tests in both diagnostic model development and validation studies.

Several key areas for improvements in reporting were identified in our study, including treatments received by participants, blinding, and study size determination. In particular, no study reported on calibration, a crucial yet often unevaluated measure of model performance [55]. We recommend assessing calibration using the calibration hierarchy described by Van Calster, et al. [56] instead of the commonly used Hosmer-Lemeshow test [57]. This avoids artificial stratification of patients into risk groups and other limitations associated with the Hosmer-Lemeshow test [55].

Studies should also strive to validate their data using stricter validation techniques. Studies with smaller sample sizes should utilise re-sampling techniques, such as bootstrapping or *k*-fold cross-validation. Studies with larger sample sizes should use a non-random split of data (e.g., by location or time) or perform external validation on independent data, for example, from a different study [25,26,58]. Validation using the same data for model development is inappropriate as it only provides apparent model performance. Similarly, validation using a random split of data, such as a ‘train-test’ split, has lower power than re-sampling techniques [25,59] and should be avoided.

In addition to the limitations in quality and reporting of AI applications, the narrow scope of applications being investigated naturally leads to fewer AI applications eventually being suitable for clinical use. While AI has been practically applied for the identification of candidate drugs for drug repurposing [60] and contact tracing [21], its application and utility for COVID-19 in clinical settings have been insignificant to date. Several studies have employed AI techniques for the detection and classification of COVID-19 images [23], however, none have been validated as a clinical diagnostic adjunct in the ED. Factors that may contribute to this lack of clinical validation include the high risk of bias within existing models [22,61], limited applicability of radiographic images for discriminating between multiple differential diagnoses, and the high prevalence of asymptomatic radiographs in patients who present soon after the onset of symptoms [62,63]. Notwithstanding the high risk of bias and poor reporting of the reviewed AI models, AI algorithms tend to produce uninterpretable “black box” predictive models, which may lead to decreased acceptability of both diagnostic and prognostic AI applications amongst clinicians and hospital administrators. Some studies [35,39,40] have attempted to overcome this by using AI techniques for feature selection and presenting the final model as a decision tree or scoring system with clearly defined input variables. However, such simplifications of AI models curtail performance and limit the utility of the final model.

The above barriers to the validation and integration of AI in clinical settings may preclude significant contribution of AI to combatting the COVID-19 pandemic in intensive care units and emergency departments in the near future. However, improvements in the development, validation, and reporting of AI applications will be critical in advancing the applicability and acceptance of these systems in clinical settings in later phases of the COVID-19 pandemic and in future global health crises. Encouragingly, leading journals such as the *Lancet* family of journals have committed to enforcing AI-specific guidelines such as CONSORT-AI and SPIRIT-AI for submissions with an AI intervention [64]. However, concerted effort is needed from the entire research community, including journals, editors, and authors, to normalise the use of these guidelines and checklists. Such changes will encourage improved development, reporting, and eventual clinical uptake of future AI applications.

### Limitations

The results from our systematic review should be considered along with the following limitations. Firstly, our search excluded non-peer-reviewed articles, such as abstracts, posters, conference proceedings, and papers from preprint servers like bioRxiv and medRxiv, which may neglect the most recent literature but ensures a baseline quality of included studies. Secondly, we may have missed some relevant articles despite using a comprehensive search strategy due to publication in journals not indexed in the searched databases and variations in terminology used to describe AI algorithms and intensive care and emergency settings. We may also have missed AI applications that were deployed without publication in scientific literature; in particular, given the intense media attention and the pressure to deploy solutions quickly, AI solutions developed by governments and industry are more likely to be published in mass media formats rather than scientific journals. Thirdly, assessment according to PROBAST and, to a lesser extent, TRIPOD reporting guidelines still rely on a degree of subjectivity, despite comprehensive explanations and elaborations. Hence, other reviewers may arrive at slightly differing results. Lastly, the unprecedented volume of research on COVID-19 has resulted in a rapidly evolving body of literature. Hence, our findings are merely descriptive of the current situation, which may change with welcome improvements and additions to the medical literature.

## 5. Conclusions

Despite widespread interest in novel technologies for the COVID-19 pandemic, our systematic review of the literature reveals that current AI applications were limited in both the range of applications and clinical applicability. Several significant issues in the development, validation, and reporting of AI applications undermine safe and effective implementation of these systems within intensive care units or emergency departments. The integration of new AI-specific reporting guidelines like CONSORT-AI and SPIRIT-AI into research and publication processes will be a vital step in creating future AI applications that are clinically acceptable in the current pandemic, future pandemics, and within the wider medical field. We also emphasise the importance of closer interdisciplinary collaboration between AI experts and clinicians.

## Figures and Tables

**Table 1:** Main study characteristics.

Author [Reference]	Study Type	Country of Study Population	Relevant Setting of Collected Data (ED, ICU, or Prehospital)	Outcome Predicted	Sample Size of Training Dataset	Sample Size of Test Dataset	Model Performance	TRIPOD Classification
Diagnostic								
Brinati, et al. [38]	Retrospective	Italy	ED	Positive COVID-19 status	279	N/A (cross-validation)	Random forest (C-index = 0.84)	1b
Fraser, et al. [39]	Prospective	Canada	ICU	Positive COVID-19 status	20	N/A (cross-validation)	Decision tree (accuracy = 98%)	1b
Vasse, et al. [40]	Retrospective	France	ED	Positive COVID-19 status	744	2390	Decision tree (Sensitivity = 60.5%, Specificity = 89.7%)	2b
Prognostic								
Abdulaal, et al. [30]	Retrospective	United Kingdom	ED	In-patient mortality	318	80	Neural network (C-index = 0.901)	2a
Assaf, et al. [31]	Retrospective	Israel	ED; ICU	Critical illness (mechanical ventilation, ICU admission, multi-organ failure, and/or death)	162	N/A (cross-validation)	Random forest (C-index = 0.93)	1b
Burdick, et al. [32]	Prospective	United States	ICU	Decompensation leading to mechanical ventilation within 24 h	49,623	197	Gradient boosting machine (C-index = 0.866)	3
Burian, et al. [33]	Prospective	Germany	ICU	ICU admission	65	N/A (cross-validation)	Random forest (C-index = 0.79)	1b
Cheng, et al. [34]	Retrospective	United States	ICU	ICU admission within 24 h	401	521	Random forest (C-index = 0.799)	2a
Durhan, et al. [41]	Retrospective	Turkey	ICU	ICU admission (software evaluates the extent of normal lung parenchyma)	90	N/A	Deep learning software (C-index = 0.944)	N/A
Jackson, et al. [35]	Retrospective	United States	ICU	Invasive mechanical ventilation	297	N/A	Fast-and-frugal decision tree (accuracy = 70%)	1a
Liang, et al. [36]	Retrospective	China	ICU	Critical illness (ICU admission, invasive ventilation, death)	1590	710	Deep learning survival Cox model (C-index = 0.852–0.967)	2b
Mushtaq, et al. [42]	Prospective	Italy	ICU	ICU admission (software evaluates the extent of lung opacity and consolidation)	697	N/A	Deep learning software based on convolutional neural networks (C-index = 0.77)	N/A
Schwab, et al. [37]	Retrospective	Brazil	ICU	ICU admission	391	167	Support vector machine (C-index = 0.98)	2a
Resource optimisation								
Belciug, et al. [43]	Retrospective	Italy	ICU	Developed a model for simulating ICU bed occupancy	N/A	N/A	Artificial immune system algorithm (no accuracy measure estimated)	N/A

COVID-19: coronavirus disease 2019, ED: Emergency Department, N/A: Not applicable, ICU: Intensive Care Unit; a: Performance of the best performing model is reported if multiple models were constructed. Only the performance on the strictest form of validation is reported. A range is given if the model was validated on multiple datasets. b: TRIPOD classification according to strictest validation used (higher values indicate stricter classification, i.e., type 3 is the strictest amongst included studies). 1a: Performance is evaluated directly on the same data; 1b: Performance and optimism of the model are evaluated using re-sampling techniques, such as bootstrapping or *k*-fold cross-validation; 2a: Model development and performance evaluation are done separately on a random split of the data, such as a train-test split; 2b: Model development and performance evaluation is done separately on a non-random split of the data by time, location, or both; 3: Model development and performance evaluation are conducted on separate data sets, for example, from different studies.

**Table 2:** PROBAST (prediction model risk of bias assessment tool) assessment of predictive modelling studies.

Author [Reference]	Risk of Bias according to PROBAST Domain				
	Participants	Predictors	Outcomes	Analysis	Overall
Diagnostic					
Brinati, et al. [38]	Low	Low	Low	High	High
Fraser, et al. [39]	Low	Unclear	Low	High	High
Vasse, et al. [40]	Low	Low	Low	High	High
Prognostic					
Abdulaal, et al. [30]	Low	Low	Low	High	High
Assaf, et al. [31]	Low	Low	Unclear	High	High
Burdick, et al. [32]	Low	Low	Unclear	High	High
Burian, et al. [33]	Low	Low	Low	High	High
Cheng, et al. [34]	Low	High	Unclear	High	High
Jackson, et al. [35]	Low	High	High	High	High
Liang, et al. [36]	Unclear	High	High	High	High
Schwab, et al. [37]	Unclear	Unclear	Unclear	High	High
Vasse, et al. [40]	Low	Low	Low	High	High

## Supplementary Materials

The following are available online at https://www.mdpi.com/article/10.3390/ijerph18094749/s1, supplementary File S1: PRISMA checklist (file format: .docx); supplementary File S2: Search strategy (file format: .docx); supplementary File S3: Modified TRIPOD checklist (file format: .docx).
