# Supplementary material for: Artificial Intelligence Applications for COVID-19 in Intensive Care and Emergency Settings: A Systematic Review
Source: Int J Environ Res Public Health. 2021 Apr 29;18(9):4749. doi: 10.3390/ijerph18094749 (PMC8125462; doi:10.3390/ijerph18094749)
Supplement: Supplementary file 1 [file ijerph-18-04749-s001.zip › ijerph-1177650-SG-SI-supplementary/Additional file 2 - Search strategy.docx]

**Supplementary material—Search strategy**

We searched PubMed, Embase, Scopus, and CINAHL to ensure a wide catchment of the medical and scientific literature. We also included IEEE Xplore and the ACM Digital Library in our search to capture more specialised research on machine learning and artificial intelligence. We combined three broad concept sets on artificial intelligence, COVID-19, and emergency or critical care settings to conduct our search. We did not include search terms for specific applications such as ‘diagnosis’, ‘prognosis’, or ‘prediction’ as we intended to capture the breadth of artificial intelligence applications available for use in the emergency and critical care settings.

**Concept sets**

- **Artificial intelligence**: (“Artificial intelligence” OR “Deep learning” OR “Machine learning” OR “AI” OR “pattern recognition” OR “Neural network” OR “Neural networks” OR “Neural networking” OR “CNN” OR “DNN” OR “RNN” OR “LSTM” OR “Long short-term memory” OR “BERT” OR “Transformer” OR “Attention model*” OR “Attention mechanism” OR “Attention mechanisms” OR “fuzzy logic” OR “Natural language” OR “Robotic*” OR “Data mining” OR “Regression” OR “nearest neighbor” OR “nearest neighbour” OR “nearest neighbors” OR “nearest neighbours” OR “kNN” OR “Decision tree” OR “Decision trees” OR “Bayes*” OR “Random forest” OR “Random forests” OR “RF” OR “Dimensionality reduction” OR “Principal component” OR “PCA” OR “Support vector” OR “SVM” OR “boost*” OR “GBM” OR “AdaBoost” OR “LightGBM” OR “XGBoost” OR “CatBoost”).
- **COVID-19**: (“COVID-19” OR “Coronavirus disease 2019” OR “2019-nCoV” OR “SARS-COV-2” OR “Severe Acute Respiratory Syndrome-2” OR “novel coronavirus”).
- **Emergency or critical care setting**: (Emergency OR “ED” OR “intensive care” OR Emergencies OR “ER” OR “EMS” OR “ICU” OR “critical care” OR “prehospital” OR “ambulance*” OR “paramedic*”).

**Scopus**

TITLE-ABS-KEY ( “Artificial intelligence” OR “Deep learning” OR “Machine learning” OR “AI” OR “pattern recognition” OR “Neural network” OR “Neural networks” OR “Neural networking” OR “CNN” OR “DNN” OR “RNN” OR “LSTM” OR “Long short-term memory” OR “BERT” OR “Transformer” OR “Attention model*” OR “Attention mechanism” OR “Attention mechanisms” OR “fuzzy logic” OR “Natural language” OR “Robotic*” OR “Data mining” OR “Regression” OR “nearest neighbor” OR “nearest neighbour” OR “nearest neighbors” OR “nearest neighbours” OR “kNN” OR “Decision tree” OR “Decision trees” OR “Bayes*” OR “Random forest” OR “Random forests” OR “RF” OR “Dimensionality reduction” OR “Principal component” OR “PCA” OR “Support vector” OR “SVM” OR “boost*” OR “GBM” OR “AdaBoost” OR “LightGBM” OR “XGBoost” OR “CatBoost” ) AND TITLE-ABS-KEY ( “COVID-19” OR “Coronavirus disease 2019” OR “2019-nCoV” OR “SARS-COV-2” OR “Severe Acute Respiratory Syndrome-2” OR “novel coronavirus” ) AND TITLE-ABS-KEY ( emergency OR emergencies OR “ED” OR “ER” OR “EMS” OR “ICU” OR “critical care” OR “intensive care” OR “prehospital” OR “ambulance*” OR “paramedic*”).

**PubMed**

(“Artificial intelligence”[Title/Abstract] OR “Deep learning”[Title/Abstract] OR “Machine learning”[Title/Abstract] OR “AI”[Title/Abstract] OR “pattern recognition”[Title/Abstract] OR “Neural network”[Title/Abstract] OR “Neural networks”[Title/Abstract] OR “Neural networking”[Title/Abstract] OR “CNN”[Title/Abstract] OR “DNN”[Title/Abstract] OR “RNN”[Title/Abstract] OR “LSTM”[Title/Abstract] OR “Long short-term memory”[Title/Abstract] OR “BERT”[Title/Abstract] OR “Transformer”[Title/Abstract] OR “Attention model*”[Title/Abstract] OR “Attention mechanism”[Title/Abstract] OR “Attention mechanisms”[Title/Abstract] OR “fuzzy logic”[Title/Abstract] OR “Natural language”[Title/Abstract] OR “Robotic*”[Title/Abstract] OR “Data mining”[Title/Abstract] OR “Regression”[Title/Abstract] OR “nearest neighbor”[Title/Abstract] OR “nearest neighbour”[Title/Abstract] OR “nearest neighbors”[Title/Abstract] OR “nearest neighbours”[Title/Abstract] OR “kNN”[Title/Abstract] OR “Decision tree”[Title/Abstract] OR “Decision trees”[Title/Abstract] OR “Bayes*”[Title/Abstract] OR “Random forest”[Title/Abstract] OR “Random forests”[Title/Abstract] OR “RF”[Title/Abstract] OR “Dimensionality reduction”[Title/Abstract] OR “Principal component”[Title/Abstract] OR “PCA”[Title/Abstract] OR “Support vector”[Title/Abstract] OR “SVM”[Title/Abstract] OR “boost*”[Title/Abstract] OR “GBM”[Title/Abstract] OR “AdaBoost”[Title/Abstract] OR “LightGBM”[Title/Abstract] OR “XGBoost”[Title/Abstract] OR “CatBoost”[Title/Abstract]) AND (“COVID-19”[Title/Abstract] OR “Coronavirus disease 2019”[Title/Abstract] OR “2019-nCoV”[Title/Abstract] OR “SARS-COV-2”[Title/Abstract] OR “Severe Acute Respiratory Syndrome-2”[Title/Abstract] OR “novel coronavirus”[Title/Abstract]) AND (Emergency[Title/Abstract] OR Emergencies[Title/Abstract] OR “ED”[Title/Abstract] OR “ER”[Title/Abstract] OR “EMS”[Title/Abstract] OR “ICU”[Title/Abstract] OR “critical care”[Title/Abstract] OR “intensive care”[Title/Abstract] OR “prehospital”[Title/Abstract] OR “ambulance*”[Title/Abstract] OR “paramedic*”[Title/Abstract]).

**IEEE Xplore** (full text searched as unable to search title or abstract simultaneously)

((“Artificial intelligence” OR “Deep learning” OR “Machine learning” OR “AI” OR “pattern recognition” OR “Neural network” OR “Neural networks” OR “Neural networking” OR “CNN” OR “DNN” OR “RNN” OR “LSTM” OR “Long short-term memory” OR “BERT” OR “Transformer” OR “Attention model*” OR “Attention mechanism” OR “Attention mechanisms” OR “fuzzy logic” OR “Natural language” OR “Robotic*” OR “Data mining” OR “Regression” OR “nearest neighbor” OR “nearest neighbour” OR “nearest neighbors” OR “nearest neighbours” OR “kNN” OR “Decision tree” OR “Decision trees” OR “Bayes*” OR “Random forest” OR “Random forests” OR “RF” OR “Dimensionality reduction” OR “Principal component” OR “PCA” OR “Support vector” OR “SVM” OR “boost*” OR “GBM” OR “AdaBoost” OR “LightGBM” OR “XGBoost” OR “CatBoost”) AND (“COVID-19” OR “Coronavirus disease 2019” OR “2019-nCoV” OR “SARS-COV-2” OR “Severe Acute Respiratory Syndrome-2” OR “novel coronavirus”) AND (Emergency OR Emergencies OR “ED” OR “ER” OR “EMS” OR “ICU” OR “critical care” OR “intensive care” OR “prehospital” OR “ambulance*” OR “paramedic*”)).

**Embase**

(‘artificial intelligence’:ab,ti OR ‘deep learning’:ab,ti OR ‘machine learning’:ab,ti OR ‘ai’:ab,ti OR ‘pattern recognition’:ab,ti OR ‘neural network’:ab,ti OR ‘neural networks’:ab,ti OR ‘neural networking’:ab,ti OR ‘cnn’:ab,ti OR ‘dnn’:ab,ti OR ‘rnn’:ab,ti OR ‘lstm’:ab,ti OR ‘long short-term memory’:ab,ti OR ‘bert’:ab,ti OR ‘transformer’:ab,ti OR ‘attention model*’:ab,ti OR ‘attention mechanism’:ab,ti OR ‘attention mechanisms’:ab,ti OR ‘fuzzy logic’:ab,ti OR ‘natural language’:ab,ti OR ‘robotic*’:ab,ti OR ‘data mining’:ab,ti OR ‘regression’:ab,ti OR ‘nearest neighbor’:ab,ti OR ‘nearest neighbour’:ab,ti OR ‘nearest neighbors’:ab,ti OR ‘nearest neighbours’:ab,ti OR ‘knn’:ab,ti OR ‘decision tree’:ab,ti OR ‘decision trees’:ab,ti OR ‘bayes*’:ab,ti OR ‘random forest’:ab,ti OR ‘random forests’:ab,ti OR ‘rf’:ab,ti OR ‘dimensionality reduction’:ab,ti OR ‘principal component’:ab,ti OR ‘pca’:ab,ti OR ‘support vector’:ab,ti OR ‘svm’:ab,ti OR ‘boost*’:ab,ti OR ‘gbm’:ab,ti OR ‘adaboost’:ab,ti OR ‘lightgbm’:ab,ti OR ‘xgboost’:ab,ti OR ‘catboost’:ab,ti) AND (‘covid-19’:ab,ti OR ‘coronavirus disease 2019’:ab,ti OR ‘2019-ncov’:ab,ti OR ‘sars-cov-2’:ab,ti OR ‘severe acute respiratory syndrome-2’:ab,ti OR ‘novel coronavirus’:ab,ti) AND (emergency:ab,ti OR emergencies:ab,ti OR ‘ed’:ab,ti OR ‘er’:ab,ti OR ‘ems’:ab,ti OR ‘icu’:ab,ti OR ‘critical care’:ab,ti OR ‘intensive care’:ab,ti OR ‘prehospital’:ab,ti OR ‘ambulance*’:ab,ti OR ‘paramedic*’:ab,ti) AND [embase]/lim.

**ACM Digital Library** (full text searched as unable to search title or abstract simultaneously)

(“Artificial intelligence” OR “Deep learning” OR “Machine learning” OR “AI” OR “pattern recognition” OR “Neural network” OR “Neural networks” OR “Neural networking” OR “CNN” OR “DNN” OR “RNN” OR “LSTM” OR “Long short-term memory” OR “BERT” OR “Transformer” OR “Attention model*” OR “Attention mechanism” OR “Attention mechanisms” OR “fuzzy logic” OR “Natural language” OR “Robotic*” OR “Data mining” OR “Regression” OR “nearest neighbor” OR “nearest neighbour” OR “nearest neighbors” OR “nearest neighbours” OR “kNN” OR “Decision tree” OR “Decision trees” OR “Bayes*” OR “Random forest” OR “Random forests” OR “RF” OR “Dimensionality reduction” OR “Principal component” OR “PCA” OR “Support vector” OR “SVM” OR “boost*” OR “GBM” OR “AdaBoost” OR “LightGBM” OR “XGBoost” OR “CatBoost”) AND (“COVID-19” OR “Coronavirus disease 2019” OR “2019-nCoV” OR “SARS-COV-2” OR “Severe Acute Respiratory Syndrome-2” OR “novel coronavirus”) AND (Emergency OR Emergencies OR “ED” OR “ER” OR “EMS” OR “ICU” OR “critical care” OR “intensive care” OR “prehospital” OR “ambulance*” OR “paramedic*”).

**CINAHL Plus**

(TI ( “Artificial intelligence” OR “Deep learning” OR “Machine learning” OR “AI” OR “pattern recognition” OR “Neural network” OR “Neural networks” OR “Neural networking” OR “CNN” OR “DNN” OR “RNN” OR “LSTM” OR “Long short-term memory” OR “BERT” OR “Transformer” OR “Attention model*” OR “Attention mechanism” OR “Attention mechanisms” OR “fuzzy logic” OR “Natural language” OR “Robotic*” OR “Data mining” OR “Regression” OR “nearest neighbor” OR “nearest neighbour” OR “nearest neighbors” OR “nearest neighbours” OR “kNN” OR “Decision tree” OR “Decision trees” OR “Bayes*” OR “Random forest” OR “Random forests” OR “RF” OR “Dimensionality reduction” OR “Principal component” OR “PCA” OR “Support vector” OR “SVM” OR “boost*” OR “GBM” OR “AdaBoost” OR “LightGBM” OR “XGBoost” OR “CatBoost” ) OR AB ( “Artificial intelligence” OR “Deep learning” OR “Machine learning” OR “AI” OR “pattern recognition” OR “Neural network” OR “Neural networks” OR “Neural networking” OR “CNN” OR “DNN” OR “RNN” OR “LSTM” OR “Long short-term memory” OR “BERT” OR “Transformer” OR “Attention model*” OR “Attention mechanism” OR “Attention mechanisms” OR “fuzzy logic” OR “Natural language” OR “Robotic*” OR “Data mining” OR “Regression” OR “nearest neighbor” OR “nearest neighbour” OR “nearest neighbors” OR “nearest neighbours” OR “kNN” OR “Decision tree” OR “Decision trees” OR “Bayes*” OR “Random forest” OR “Random forests” OR “RF” OR “Dimensionality reduction” OR “Principal component” OR “PCA” OR “Support vector” OR “SVM” OR “boost*” OR “GBM” OR “AdaBoost” OR “LightGBM” OR “XGBoost” OR “CatBoost” )) AND (TI ( “COVID-19” OR “Coronavirus disease 2019” OR “2019-nCoV” OR “SARS-COV-2” OR “Severe Acute Respiratory Syndrome-2” OR “novel coronavirus” ) OR AB ( “COVID-19” OR “Coronavirus disease 2019” OR “2019-nCoV” OR “SARS-COV-2” OR “Severe Acute Respiratory Syndrome-2” OR “novel coronavirus” )) AND (TI ( Emergency OR Emergencies OR “ED” OR “ER” OR “EMS” OR “ICU” OR “critical care” OR “intensive care” OR “prehospital” OR “ambulance*” OR “paramedic*” ) OR AB ( Emergency OR Emergencies OR “ED” OR “ER” OR “EMS” OR “ICU” OR “critical care” OR “intensive care” OR “prehospital” OR “ambulance*” OR “paramedic*” )).
