# Supplementary material for: Artificial Intelligence Applications for COVID-19 in Intensive Care and Emergency Settings: A Systematic Review
Source: Int J Environ Res Public Health. 2021 Apr 29;18(9):4749. doi: 10.3390/ijerph18094749 (PMC8125462; doi:10.3390/ijerph18094749)
Supplement: Supplementary file 1 [file ijerph-18-04749-s001.zip › ijerph-1177650-SG-SI-supplementary/Additional file 3 - Modified TRIPOD checklist.docx]

**S1 Table.** Adjusted TRIPOD checklist for reporting quality assessment.

| **Section/Topic** | **Item** | **Checklist Item** |
| --- | --- | --- |
| **Methods** | | |
| Source of data | 4a | Describe the study design or source of data (e.g., randomized trial, cohort, or registry data), separately for the development and validation data sets, if applicable. |
|  | 4b | Specify the key study dates, including start of accrual; end of accrual; and, if applicable, end of follow-up. |
| Participants | 5a | Specify key elements of the study setting (e.g., primary care, secondary care, general population) including number and location of centres. |
|  | 5b | Describe eligibility criteria for participants. |
|  | 5c | Give details of treatments received, if relevant. |
| Outcome | 6a | Clearly define the outcome that is predicted by the prediction model, including how and when assessed. |
|  | 6b | Report any actions to blind assessment of the outcome to be predicted. |
| Predictors | Adjusted 7a | Clearly define all predictors used in developing or validating the **AI or ML** model, including how and when they were measured. |
|  | 7b | Report any actions to blind assessment of predictors for the outcome and other predictors. |
| Sample size | 8 | Explain how the study size was arrived at. |
| Missing data | 9 | Describe how missing data were handled (e.g., complete-case analysis, single imputation, multiple imputation) with details of any imputation method. |
| Statistical analysis methods | 10a | Describe how predictors were handled in the analyses. |
|  | Adjusted 10b | Specify type of model, all model-building procedures (including any predictor selection, **hyperparameter selection if needed**), and method for internal validation. |
|  | 10c | For validation, describe how the predictions were calculated. |
|  | 10d | Specify all measures used to assess model performance and, if relevant, to compare multiple models. |
| Risk groups | 11 | Provide details on how risk groups were created, if done. |
| Development vs. validation | 12 | For validation, identify any differences from the development data in setting, eligibility criteria, outcome, and predictors. |
| **Results** | | |
| Participants | 13a | Describe the flow of participants through the study, including the number of participants with and without the outcome and, if applicable, a summary of the follow-up time. A diagram may be helpful. |
|  | 13b | Describe the characteristics of the participants (basic demographics, clinical features, available predictors), including the number of participants with missing data for predictors and outcome. |
| Model development | 14a | Specify the number of participants and outcome events in each analysis. |
|  | 14b | If done, report the unadjusted association between each candidate predictor and outcome. |
| Model specification | Adjusted 15a | Present the full prediction model to allow predictions for individuals (**i.e. links to the final model online (coding of predictors, code and final parameters/coefficients, and with the architecture described in full in the article)**). |
|  | 15b | Explain how to the use the prediction model. |
| Model performance | Adjusted 16a | Report **discriminative** performance measures (with CIs) for the prediction model. |
|  | Adjusted 16b | **Report calibration performance measures (with CIs) for the prediction model.** |
